# Exposure to Nonhuman Primates in Rural Cameroon

**DOI:** 10.3201/eid1012.040062

**Published:** 2004-12

**Authors:** Nathan D. Wolfe, A. Tassy Prosser, Jean K. Carr, Ubald Tamoufe, Eitel Mpoudi-Ngole, J. Ndongo Torimiro, Matthew LeBreton, Francine E. McCutchan, Deborah L. Birx, Donald S. Burke

**Affiliations:** * Johns Hopkins Bloomberg School of Public Health, Baltimore, Maryland, USA;; † Henry M. Jackson Foundation, Rockville, Maryland, USA;; ‡ Johns Hopkins Cameroon Program, Yaoundé, Cameroon;; § Army Health Research Center (CRESAR), Yaoundé, Cameroon;; ¶ Walter Reed Army Institute of Research, Rockville, Maryland, USA

**Keywords:** Primate Diseases, Zoonoses, Ecology, Cross-species transmission, Hunting, Bushmeat, Central Africa, research

## Abstract

Exposure to nonhuman primates has led to the emergence of important diseases, including Ebola hemorrhagic fever, AIDS, and adult T-cell leukemia. To determine the extent of exposure to nonhuman primates, persons were examined in 17 remote villages in Cameroon that represented three habitats (savanna, gallery forest, and lowland forest). Questionnaire data were collected to assess whether persons kept wild animal pets; hunted and butchered wild game; had experienced bites, scratches, or injuries from live animals; or had been injured during hunting or butchering. While all villages had substantial exposure to nonhuman primates, higher rates of exposure were seen in lowland forest sites. The study demonstrates that exposure is not limited to small groups of hunters. A high percentage of rural villagers report exposure to nonhuman primate blood and body fluids and risk acquiring infectious diseases.

## Abstract 2

A high percentage of rural villagers are exposed to blood of nonhuman primates and risk acquiring infectious diseases.

Closely related species generally share susceptibility to the same groups of microorganisms (*1*). The anthropoid primates, which include humans, and to a lesser degree simian primates share broadly similar physiologic and genetic characteristics and thus susceptibility to many viruses, bacteria, fungi, protozoa, helminths and ectoparasites (*2**,**3*). Members of the family *Hominidae*, which includes humans, chimpanzees, bonobos, and gorillas, share an even greater similarity in susceptibility to microorganisms (*3*). Our closest relatives, chimpanzees and, most likely, bonobos, share with us the potential for infection with virtually the same set of microorganisms.

A range of activities involves direct contact between humans and nonhuman primates and allows for the transmission of microorganisms. Such behavior can facilitate transmission of microorganisms from nonhuman primates to humans (*4*), with consequences for human health, as well as from humans to nonhuman primates, with consequences for wildlife conservation (*5*). Care for captive nonhuman primates has lead to the transmission of a range of infections, including simian foamy virus (*6*), herpesvirus B (HBV) (*7*), primate malaria (*8*), and tuberculosis (*9*). Nonhuman primate ecotourism (e.g., gorilla watching) has been associated with the possible transmission from humans to nonhuman primates of diseases that include scabies (*Sarcoptes scabiei*) (*10*), intestinal parasites (*11*), and measles (*12*). Laboratory handling of tissues or fluids of nonhuman primates has lead to transmission of a range of infections to humans, including simian immunodeficiency virus (SIV) (*13*) and SV40, which was subsequently distributed through oral polio vaccine to millions of people (*14*). Additionally, keeping nonhuman primate pets has been linked to transmission of a variety of microorganisms (*15*). Finally, hunting and butchering nonhuman primates have been linked to the transmission of Ebola (*16**,**17*), monkeypox (*18*), and simian foamy virus (*19*). Because of the broad range of fluid and tissue types involved with hunting and butchering, this mechanism of transmission may be particularly important in cross-species transmission (*1*), although other behavior, such as wildlife necropsy, has similar risks (*20*).

A number of important human diseases, including AIDS (HIV), adult T-cell leukemia (HTLV-1) and malaria (*Plasmodium* spp.), are believed to have emerged as the results of ancient or contemporary cross-species transmission from nonhuman primates. While the emergence of malaria was presumably the result of vector-borne transmission, the mechanisms which led to the emergence of HIV and HTLV remain unknown. One of the current primary hypotheses to explain the origins of HIV is that hunting and butchering nonhuman primates led to cross-species transmission (*21**,**22*). This hypothesis is strengthened by recent evidence suggesting that hunted nonhuman primates have a high rate of SIV infection (*23*) and evidence of hunting-associated cross-species transmission of a nonhuman primate retrovirus, simian foamy virus, in central African hunters (*19*).

While some groups are at risk for contact with nonhuman primates, the frequency of behavior involving exposure to nonhuman primates remains largely unknown. The objective of the present study was to use behavioral tools to examine the frequency and extent of exposure to nonhuman primates among persons living in rural village sites in a region of high primate biologic diversity.

## Materials and Methods

### Participants

**Figure 1:**
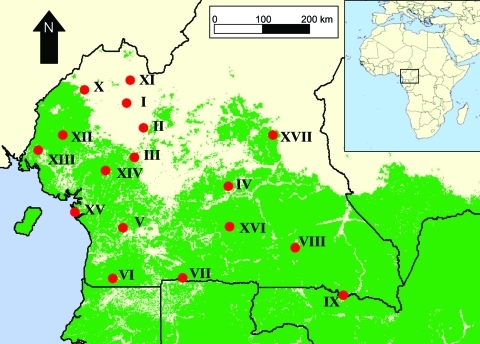
Map of study sites in southern Cameroon in relation to the distribution of lowland tropical forest in central Africa (in green).

Seventeen village sites in Cameroon were selected for this study (Table 1, Figure 1). Sites were selected in highly rural areas, often at the end of small, unpaved roads. Sites were chosen to obtain different habitats, including 2 savanna sites, 2 gallery forest sites, and 13 lowland forest sites, and based on their proximity to regions supporting wild game populations. The sites selected are all in the southern part of Cameroon, a region that includes extensive lowland rainforest (Figure 1). The 17 sites include 2 in each of the Southwest, Northwest, West, Littoral, and Central Provinces, 3 in the South Province, and 4 in the East Province. All 17 sites in the study participated in commercial sales of hunted wild game.

**Table 1:** Characteristics of the 17 rural Cameroonian villages

Site no.	Latitude	Longitude	Altitude (m)	Habitat type	River basin (tributary)	Language group	Located at end of road
I	5.8°	10.7°	1,180	Savanna	Sanaga (Noun)	Shu-pamen	Yes
II	5.3°	11.0°	730	Gallery forest	Sanaga (Mbam)	Shu-pamen	No
III	4.8°	10.8°	800	Lowland forest	Wouri/Sanaga	Tunen	Yes
IV	4.2°	12.7°	680	Lowland forest	Nyong	Beti-fang	Yes
V	3.4°	10.6°	120	Lowland forest	Nyong/Lokoundje	Kwasio	No
VI	2.3°	10.4°	400	Lowland forest	Ntem	Beti-fang	Yes
VII	2.4°	11.8°	560	Lowland forest	Ntem	Beti-fang	No
VIII	2.2°	14.1°	630	Lowland forest	Congo (Dja)	Kawzime	No
IX	2.4°	15.0°	330	Lowland forest	Congo (Dja)	Mpo	Yes
X	6.1°	9.8°	1,500	Gallery forest	Calabar/Niger (Cross)	Esimbi	Yes
XI	6.3°	10.8°	1,700	Savanna	Niger	Limbum	Yes
XII	5.2°	9.4°	350	Lowland forest	Calabar (Cross)	Kenyang	No
XIII	4.9°	8.9°	100	Lowland forest	Ndian	Oroko	No
XIV	4.5°	10.3°	200	Lowland forest	Wouri (Dibamba)	Bassa	No
XV	3.7°	9.7°	0	Lowland forest	Sanaga	Duala	Yes
XVI	3.4°	12.7°	650	Lowland forest	Congo (Dja)	Beti-fang	Yes
XVII	5.2°	13.6°	640	Gallery forest	Sanaga	Gbete	Yes

Studies were conducted in the context of a community-based HIV prevention campaign designed to provide information and decrease transmission. Participation in the study was voluntary. Persons who participated in the HIV prevention campaign were asked if they would like to hear more about a research study, and the study was described to those who were interested. Study description, the informed consent procedure, and questionnaire administration were all done orally in English or French, which are widely spoken as second languages in the study villages. Participants were offered compensation approximately equivalent to 1 day of work, since participation often precluded farm work on that day. The study protocol was approved by the Johns Hopkins Committee for Human Research, the Cameroon National Ethical Review Board, and the HIV Tri-Services Secondary Review Board. In addition, a single project assurance was obtained from the Cameroonian Ministry of Health and accepted by the National Institutes of Health Office for Protection from Research Risks.

### Behavioral Data

After the informed consent process, participants were asked to respond to a behavioral questionnaire. The questionnaire was administered individually by trained interviewers, without regard to the sex of the participant. The questionnaire, which was linked, was designed to provide basic demographic information as well as information on behavior either directly or indirectly associated with exposure to the blood or body fluids of nonhuman primates. The questionnaire was pretested in Cameroon before use. Behaviors considered indirectly associated with exposure to blood or body fluids include butchering, hunting, and keeping pets. Behavior considered to be directly associated with exposure to blood or body fluids includes having been scratched or bitten by a nonhuman primate or having been injured while hunting or butchering. Following pretests, locally appropriate taxonomic categories were derived from accepted local terms for animals. For example, "monkey" was identified as an appropriate taxonomic category, and participants were not asked to distinguish between monkey species. Other taxonomic categories used include chimpanzee, gorilla, and rodent. Rodents were included because they are perhaps the most commonly hunted and eaten type of forest animal and serve as a useful comparison with nonhuman primates. Participants were asked to identify which of these four taxa they had consumed, hunted, butchered, or kept as pets. Participants were asked to estimate their monthly frequency of consumption of each of the wild taxa and of the wild game overall. Participants were also asked to report on direct contact with wild taxa, including the taxa involved, having been scratched or bitten, or having been injured while hunting or butchering.

## Results

A total of 3,971 persons were interviewed. Both men and women participated and were represented approximately equally in the sites, with 46.3% female participants and 53.7% male participants. These aggregate results were similar to those found within the sites. Participants' ages ranged from 16 to 97 years. Participants were not equally distributed with regards to age; younger age groups were more represented. Participants were classed into four age groups: 16–30 years, 31–45 years, 46–60 years, and >60 years. Ages 16–30 made up 42% of participants, ages 31–45 made up 27% of participants, ages 46–60 made up 21% of participants, and ages >60 made up 10% of participants.

**Figure 2:**
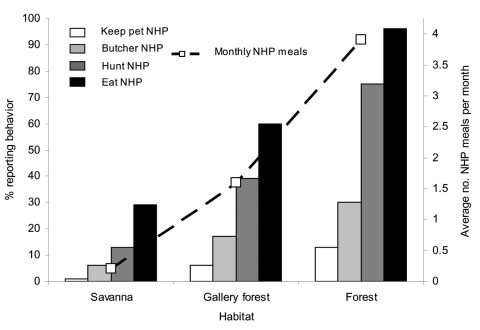
Percentage of participants in rural villages reporting exposure to wild game (monkeys, chimpanzees, and gorillas combined) by keeping pets, hunting, butchering, and eating, with average monthly frequency of wild game meal consumption for all species examined.

Participants reported having kept pets in all four taxonomic categories. Monkeys were kept as pets more frequently than other types of wild animals. The overall percentage of participants keeping pets from all sites combined was 0.6% keeping gorillas, 1.5% keeping chimpanzees, 9.9% keeping monkeys, and 1.8% keeping rodents. Sites in the study differed in their tendency to keep pets; persons in lowland sites reported keeping pets more frequently than people in gallery and savanna sites did (Figure 2).

**Figure 3:**
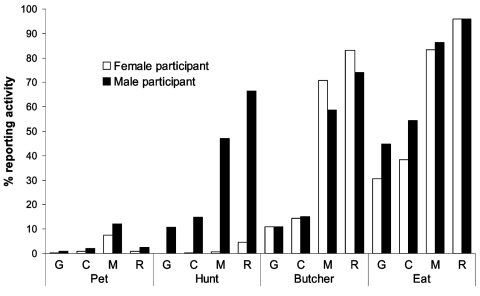
Percentage of male and female participants reporting exposure to wild game taxa (gorilla [G], chimpanzee [C], monkey [M], and rodent [R]) through keeping pets, hunting, butchering, and eating.

In addition to keeping wild animal pets, three additional contact-associated activities were examined in this study, including hunting, butchering, and eating. Participants in all sites reported having hunted, butchered, and eaten animals from the four wild game taxa examined in this study. A higher percentage had eaten wild game than had butchered wild game, and a higher percentage had butchered wild game than hunted wild game (Figure 2). Hunting, butchering, and eating wild game were more common in forest sites than in other sites. For hunting, butchering, and eating, participants in the study had greater contact with rodents and monkeys than chimpanzees and gorillas (Figure 3).

While no significant departure was seen from expected proportions of women and men reporting eating of wild game of all types (χ^2^ = 0.046, nonsignificant) or of rodents (χ^2^ = 0.001, nonsignificant), fewer women than expected and more men than expected reported eating monkeys (χ^2^ = 6.762, p < 0.01), chimpanzees (χ^2^ = 102.216, p < 0.001), and gorillas (χ^2^ = 0.046, p < 0.001). Of persons reporting hunting nonhuman primates, a higher proportion than expected by chance were men (53.7% expected, 98.7% observed) and lower proportion than expected were women (46.3% expected; 1.3% observed) (χ^2^ = 1,119.130, p < 0.001). However, of participants reporting butchering nonhuman primates, a significantly higher proportion than expected were women (46.3% expected, 50.9% observed) and a lower proportion than expected were men (53.7% expected, 49.1% observed) (χ^2^ = 61.376, p < 0.001). The average number of nonhuman primate meals differed between the three habitat types (F = 201.273, p < 0.001): it was significantly higher in lowland forest than in gallery forest (Bonferroni posthoc test p < 0.001) and significantly higher in gallery forest than in savanna (Bonferroni posthoc test p < 0.001) (Figure 2).

Data were also examined for evidence of direct contact with nonhuman primate blood and body fluids. Two types of evidence for direct exposure were examined: self-reports of scratches or bites from live nonhuman primates and self-reports of injuries involving body fluids associated with hunting and butchering nonhuman primates (Table 2). Injuries associated with hunting and butchering occurred in 14 of the 17 villages and in all three habitation types, with a total of 1.67% of participants reporting such injuries (Table A1). Scratches or bites from nonhuman primates occurred in 14 villages in gallery forest and forest but did not occur in savanna sites. A total of 2.64% of participants reported such injuries. Of the participants who reported direct contact with nonhuman primate blood and body fluids through scratches, bites, or injuries, 91.2% reported butchering nonhuman primates, 73.0% reported hunting nonhuman primates, and 43.1% reported keeping a nonhuman primate as a pet. Men made up 82.5% of participants reporting direct contact, and women made up 17.5%. Most reports of direct contact involved monkeys (73.7%), although some direct contact was reported with gorillas (16.7%) and chimpanzees (9.6%).

**Table 2:** Frequency of persons in 17 Cameroonian villages reporting direct contact with nonhuman primate body fluids

Village location	Persons reporting direct nonhuman primate body fluid contact	
	Scratch or bite, n (%)	Injury during hunting or butchering, n (%)
Savanna (N = 364)	0 (0)	2 (0.55)
Gallery forest (N = 564)	8 (1.42)	7 (1.24)
Lowland forest (N = 3,043)	97 (3.19)	57 (1.87)
Overall (N = 3,971)	105 (2.64)	66 (1.67)

## Discussion

Hunting nonhuman primates is a biologically ancient behavior that we share with our closest living relatives, the chimpanzees (*24*). Human hunting techniques and patterns, however, have changed substantially in contemporary times. During the 20th century, firearms increased the efficiency and frequency of hunting. Both subsistence and commercial hunting with wire snares and firearms are widespread activities throughout the forests of central Africa (*1**,**25**,**26*). In addition, road networks and increasing opportunities for transporting hunted game have led to an increase in sales and the rate of hunting (*27*).

Hunting and butchering nonhuman primates has been linked to the emergence of infectious disease (*1**,**4*). Hunting a red colobus (*Procolobus badius oustaleti*) has been implicated in a localized epidemic of monkeypox that continued for four generations of human-to-human contact (*18*). In addition, an outbreak of Ebola hemorrhagic fever in Mayibout, Gabon, in January 1996 was linked to butchering and eating a chimpanzee that had been found dead; 29 of 37 identified cases involved exposure to the chimpanzee (*16*). A number of subsequent epidemics in Gabon and Congo have also been linked to hunting and butchering apes (*17*).

Not only humans are at risk for diseases transmitted from nonhuman primates through hunting and butchering. Chimpanzees are regular hunters of monkeys and other forest vertebrates, and a study of Ebola hemorrhagic fever among chimpanzees in the Tai forests showed that the primary risk factor for contracting Ebola among wild chimpanzees was hunting behavior, which showed a stronger association with infection than other acknowledged risk factors, such as "touching dead bodies" (*28*).

More recent research suggests that hunting and butchering nonhuman primates resulted in the emergence of HIV (i.e., after cross-species transmission of SIV and subsequent spread) (*21**,**22*). While reconstructing the history of viral emergence is a substantial challenge, one possibility is that transmission of SIV associated with hunting and butchering is an ongoing process and that contemporary hunters may yet be found with SIV infection. This hypothesis has been strengthened by recent evidence suggesting that hunted nonhuman primates have a high rate of SIV infection (*23*) and evidence of hunting-associated cross-species transmission of another nonhuman primate retrovirus, simian foamy virus (*19*).

These results show that at least some rural villagers have a high level of exposure to nonhuman primates. While officially hunting of wild animals is forbidden, it is nonetheless widely accepted and permitted for personal use, so while the data may contain some bias, we do not feel that it is substantial. Our results show butchering to be the most common activity associated with contact with nonhuman primate blood and body fluids. More than 60% of the participants in the study reported having butchered a nonhuman primate, compared with ≈30% of participants who had hunted nonhuman primates. The higher frequency of persons reporting butchering as compared to hunting is expected, since those who hunt will often participate in some sort of butchering, generally including some preparation of wild game (e.g., disembowelment). Approximately 11% of the persons in the study reported keeping nonhuman primate pets. Because pets are usually young, the prevalence of chronic diseases in this population may be less than that among adult prey to which hunters and butchers are exposed. Nevertheless, because of the potential for regular contact with pet animals, even a low frequency of infections among pets may be important.

Villages from different habitats differed with regards to their reported exposure activities (Figure 2). Reported monthly consumption was significantly higher in the lowland forest sites. This finding may be due to the higher density and diversity of wildlife located close to these regions. Men and women both had high levels of contact with primate body fluids (Figure 3). While men were more likely than women to hunt wild animals, women were more likely than men to butcher. Because of differential participation in risk activities by men and women, these data suggest that gender-based interventions may be appropriate to decrease potential exposures to nonhuman primate blood and body fluids in central Africa.

The number of persons who reported direct contact with nonhuman primate blood or body fluids through scratches and bites from live primates or injuries during hunting and butchering was low. A total of 66 participants reported being in contact with nonhuman primate blood and body fluids through an injury associated with hunting or butchering wild game (Table A1). These persons often had multiple risk factors associated with nonhuman primate contact. Most of these persons reported a history of hunting nonhuman primates; eight persons reported only butchering. Results suggest that injuries associated with butchering may be less frequent than those associated with hunting, although injuries associated with butchering may be less severe and therefore less memorable than injuries associated with hunting and therefore underreported.

The results of this study show that contact with nonhuman primates, through both keeping wild animal pets and hunting and butchering nonhuman primates, is not confined to a small segment of the rural population. Rural central Africans are more highly exposed to microorganisms present in nonhuman primates than was previously considered. Persons in industrialized countries who have regular contact with nonhuman primates, such as laboratory workers, are known to risk contracting infectious diseases from nonhuman primates, such as herpesvirus B, SIV, and simian foamy virus. These occupationally exposed persons are the subject of extensive public health interventions, aimed at controlling zoonotic transmission (*29**,**30*). While poor rural villages depend on wild game for animal protein, education on the risks associated with contact with nonhuman primate blood is an essential step for these communities, as is work to develop economic alternatives to hunting. Studies in such villages can provide further insights into behavioral links with disease emergence. Behavioral interventions aimed at decreasing exposure to nonhuman primates in villages with high exposure rates may provide an opportunity to prevent both zoonosis on an individual level, as well as emergence events that have the potential for global effects.

## Appendix

**Table A1:** Basic demographic data and risk profiles are presented for the 66 persons reporting injuries associated with hunting or butchering monkeys (Mo), chimpanzees (C), or gorillas (Go). Hunting techniques include gun (G), wire snare (W), hand (H), and machete (M). Persons reporting hunting as a top occupation reported hunting either to sell or for home use.

ID	Site	Habitat	Age	Sex	Injury	Hunting technique	Hunting as top occupation	Hunts	Butchers	Keeps as pet
CAM0057BA	I	Savanna	58	M	Mo	G, W, H, M	No	Mo	Mo	–
CAM2887HA	XVII	Gallery forest	24	M	Go	G, W, M	No	Mo, C, Go	Mo, C, Go	Mo
CAM2940HA	XVII	Gallery forest	39	F	Mo	–	No	–	Mo, C	–
CAM3042HA	XVII	Gallery forest	74	M	Go	W, M	No	Mo, C, Go	Mo, C, Go	–
CAM3076HA	XVII	Gallery forest	33	M	Mo	W, M	No	Mo, C	Mo, C	Mo
CAM3083HA	XVII	Gallery forest	64	M	C	G, W	No	Mo, C, Go	–	–
CAM1601LE	VIII	Forest	18	M	Mo	G, W, H, M	No	Mo	Mo	–
CAM1603LE	VIII	Forest	22	M	Mo	G, W, H, M	No	Mo	Mo	–
CAM1616LE	VIII	Forest	30	M	Mo	G, W, H, M	No	Mo, C, Go	Mo, C, Go	–
CAM1654LE	VIII	Forest	42	M	Mo	G, W, O	No	Mo	–	–
CAM1695LE	VIII	Forest	31	M	Mo	G, W, H, M	No	Mo, C	Mo, C	Mo
CAM1716LE	VIII	Forest	29	F	Mo	–	No	–	Mo, C, Go	–
CAM1739LE	VIII	Forest	25	M	Mo	G, W	No	Mo, C, Go	Mo	Mo
CAM2382LE	VIII	Forest	30	M	Mo	–	No	Mo	Mo, C	–
CAM2478LE	VIII	Forest	33	M	Go	W, M	No	Mo	Mo, C, Go	–
CAM2501LE	VIII	Forest	37	M	Mo	G, W, M	No	Mo, Go	Mo, Go	Mo
CAM0136MA	II	Gallery forest	22	M	C	G, H, M	To sell	Mo	Mo, C	–
CAM0147MA	II	Gallery forest	45	M	Mo	W, H, M	No	Mo	Mo	–
CAM3618MB	XI	Savanna	20	F	Mo	–	No	–	Mo	–
CAM3138MN	XII	Forest	28	M	Mo	G, W	No	Mo, C, Go	Mo	Mo
CAM3171MN	XII	Forest	23	M	Mo	G	No	Mo, C, Go	Mo	–
CAM3192MN	XII	Forest	18	F	Mo	–	No	–	Mo	–
CAM0955MO	IX	Forest	50	M	Mo	G, W, H	No	Mo, C	Mo, C	Mo
CAM0978MO	IX	Forest	29	M	Mo	G	No	Mo, Go	Mo, Go	–
CAM0979MO	IX	Forest	30	M	Mo	G	No	Mo	Mo	–
CAM1005MO	IX	Forest	16	M	Mo	G, W	No	Mo	Mo	–
CAM1025MO	IX	Forest	17	M	Mo	G, W	No	Mo	Mo	–
CAM1051MO	IX	Forest	60	M	Go	G, W	No	Mo, C, Go	Mo, C, Go	–
CAM1501MV	VII	Forest	60	M	Go	G, W, H, M	No	Mo	Mo, C, Go	–
CAM1564MV	VII	Forest	40	M	Mo	G, W, H, M	No	Mo, C, Go	Mo, C, Go	Mo
CAM0009ND	III	Forest	51	M	Mo	W, H, M	No	Mo	Mo	–
CAM0032ND	III	Forest	23	M	Mo	G, W	Yes	Mo, C	Mo	Mo
CAM0038ND	III	Forest	24	M	Mo	G, W	To sell	Mo, C	Mo, C	Mo
CAM0053ND	III	Forest	28	M	Mo	G, W	No	Mo, C, Go	Mo, C, Go	Mo
CAM0078ND	III	Forest	43	F	Mo	–	No	–	Mo	–
CAM0142ND	III	Forest	60	M	Mo	G, W	No	Mo	Mo	–
CAM0169ND	III	Forest	55	M	Mo	G, W, H	No	Mo, C	Mo, C	Mo
CAM0174ND	III	Forest	44	M	Mo	G, W, H	No	Mo, C	Mo, C	Mo
CAM0194ND	III	Forest	35	M	Mo	G, W, H	No	Mo	Mo, C	Mo
CAM2085ND	III	Forest	41	M	Mo	G, W	No	Mo	Mo	Mo
CAM2659ND	III	Forest	42	M	Mo	G, H	No	Mo, C	Mo, C	Mo
CAM2692ND	III	Forest	28	M	Mo	G, W, H	No	Mo	Mo	Mo
CAM1177NG	V	Forest	30	M	Mo	G, W, H	To sell	Mo, C, Go	Mo, C, Go	Mo
CAM1242NG	V	Forest	59	M	Mo	W	No	Mo	Mo	Mo
CAM1281NG	V	Forest	72	M	Mo	W, H, M	No	Mo	Mo	–
CAM1303NG	V	Forest	74	M	Mo	W, H, M	No	Mo, C, Go	Mo, C, Go	Mo
CAM1311NG	V	Forest	31	M	Mo	–	No	–	Mo	–
CAM1322NG	V	Forest	56	M	Mo	G, W, H, M	No	Mo	–	–
CAM1347NG	V	Forest	33	M	Mo	G, W, H, M	No	Mo, C	Mo	Mo
CAM1364NG	V	Forest	44	M	Mo	G, W	No	Mo, C	Mo	–
CAM0003NY	VI	Forest	32	M	Mo	G, W, H, M	To sell	Mo, C	Mo, C	–
CAM0013NY	VI	Forest	60	M	Mo	W	No	Mo	Mo	Mo
CAM0017NY	VI	Forest	27	M	Mo	G, W	No	Mo	Mo, C, Go	Mo
CAM0047NY	VI	Forest	32	M	C, Ho	G, W	No	Mo, C, G	Mo, C, Go	C
CAM0092NY	VI	Forest	38	M	Go	G, W, H, M	No	Mo, C, Go	Mo, C, Go	Mo
CAM0100NY	VI	Forest	28	M	Go	G, W, H, M	No	Mo, C, Go	Mo, C, Go	Mo, C
CAM0165NY	VI	Forest	53	M	Mo	W	No	Mo	Mo	–
CAM0169NY	VI	Forest	69	M	Mo	G, W	No	Mo, C, Go	Mo, C, Go	–
CAM0190NY	VI	Forest	47	F	Mo	–	No	–	Mo	–
CAM2269SA	IV	Forest	62	M	Mo	G, W	No	Mo, C, Go	Mo, C, Go	–
CAM3738SO	XVI	Forest	33	M	C	W	No	Mo, C	Mo, C	C
CAM3793SO	XVI	Forest	20	M	Mo	G, W	No	Mo	Mo	–
CAM4321SO	XVI	Forest	32	F	Mo	–	No	–	Mo	–
CAM4340SO	XVI	Forest	20	M	Mo	G, W	For home	Mo	Mo	–
CAM2764YI	XIV	Forest	60	M	Mo	W, M	No	Mo	Mo	–
CAM4157YI	XIV	Forest	40	M	Mo	W	No	Mo	Mo	Mo
